# Case of Tumour on the Tongue, with Cursory Observations on the Use of the Carbonate of Iron in Carcinomatous and Phagedenic Ulcerations

**Published:** 1810-08

**Authors:** R. Hall

**Affiliations:** London


### *Case of Tumour on the Tongue, with cursory Observations on the Use of the Carbonate of Iron in Carcinomatous and Phagedenic Ulcerations.* By R. Hall, M. D. London.

La vraie experience est l'experience generale, qui resulte des observations particuliéres de tous les lieux et de tous les temps.

THE want of a clear and plain definition of cancer has frequently been a subject of much regret among the practitioners of the healing art. Many writers have accordingly endeavoured to supply this desideratum, but hitherto with little success; for though we find various definitions given of it in nosological systems, all of them, it may be affirmed, are imperfect and insufficient for the purpose of accurate distinction; since they still leave us in ignorance of what constitutes its essence, or essential character. Hence, scarcely any term has been more vaguely used than that of cancer; some applying it indiscriminately to all inveterate and corroding ulcers, in whatever part situated, which resist the common methods of cure; while others, on the contrary, confine it merely to indurated, and unequally enlarged glands terminating in ulceration. If, however, through our ignorance of the distinctive nature, or essential difference of cancer, a perfect definition be yet unattainable, it were much to be wished, that we possessed such a competent description of the disease, as might enable practitioners readily to discriminate its various forms, and to distinguish it from affections to which it bears the greatest affinity. For it must be obvious that, till some practical distinction be obtained, or, in other words, till precise ideas be annexed to the term cancer, practitioners in particular, who have not had much experience in this disorder, may readily confound it with other maladies, which, though apparently similar, are yet essentially different, and thus be led to the adoption of a practice which they would not otherwise have pursued.

From the want of a criterion, or some unequivocal diagnostic sign of cancer, it must likewise be ascribed, that so many impositions are daily practised on the ignorant and credulous public, by nostrum-mongers and others, from motives of cupidity and sordid views of self interest, and that various cases have been related, in which cures are confidently said to have been performed, though every person conversant with the nature and character of cancer knows how unavailing have proved all the methods hitherto proposed for the cure of this intractable disorder.

Hence

Hence likewise, we must necessarily receive, with a great degree of scepticism, the favourable reports of any new remedy suggested for the cure, or even alleviation of so hopeless a malady, however respectable the authority on which it is recommended. Nor can such scepticism, we think, appear unreasonable to any one, who reflects that, though innumerable remedies have been celebrated, at different periods, for their virtues in cancer, yet, at this moment, we know no more of its cure than was known in the days of Hippocrates and Galen.

Notwithstanding these impressions, we entered on the perusal of Mr. Carmichael's Treatise, in which the carbonate of iron is proposed as a remedy in cancer, with a considerable degree of interest, because, though attempts to cure it have hitherto proved unsuccessful, we did not consider it impossible, that others might be more fortunate, or more sagacious, than their predecessors.

Mr. Carmichael, it appears, was first induced to employ this substance in carcinomatous affections, in consequence of adopting the hypothesis, that cancer, like hydatids, is a parasitical animal endowed with independent vitality.

This belief in the animalcular nature of cancer obviously led him to the consideration, that as iron was very effectual in destroying intestinal worms, so it would prove equally destructive to other animals of a parasitical nature. The arguments, which he adduces in support of the above opinion, are chiefly founded on a loose analogy, or fancied resemblance between the structure of cancer and that of the polypus and other zoophytes; on the gratuitous assumption, that cancerous affections principally occur in parts which possess the lowest degree of vitality, and that such parts are particularly favourable to the lodgment and growth of parasitical animals.

Though the hypothesis which ascribes to carcinoma an hydatid or animalcular existence, appear, in our estimation, not less fanciful and extravagant than the arguments adduced in its support are vague and inconclusive, yet candour and philosophy suggest the propriety of suspending our judgment, as to the practice he recommends, till it has been submitted to a fair and liberal trial. In the mean time, we may be permitted to express our doubts respecting the true nature of the cases, in which this remedy proved successful. Exclusive of other circumstances, the quick relief experienced, and the rapidity with which the disease yielded to this mode of treatment, so contrary to what usually happens happens in genuine cancer, cannot fail to excite such a suspicion in the mind of any one in the least acquainted with the history and progress of this formidable malady. This suspicion is still farther strengthened by some recent reports which, if well founded, must lead us to fear, that its fate will be similar to that of the solanum, cicuta, &c, which, it is well known, though once equally extolled, no longer hold the same place in the opinions of medical men as formerly.

But whatever may be our doubts respecting the specific powers of iron in genuine cancer, we think it cannot be denied, from the cases adduced, that it has in many instances contributed, particularly when assisted by the exhibition of appropriate internal medicines, to the cure of inveterate and phagedenic ulcers, even though these had existed for a considerable length of time, and bafled various remedies.

We lately took occasion to remark, in the Medical and Physical Journal for November, 1808, page 401, that an ointment, formed of the rust of iron with citric acid and hogslard, had long ago been employed in the French West India Islands, and was considered as a very effectual remedy in cases of yaws, even when deemed otherwise incurable.

As a farther proof of its utility, it may not be here improper to relate the following instance of its beneficial effects, in what was considered, by several practitioners, as a carcinomatous affection of the tongue.

Mr. Clunes, a taylor in Stanhope Street, about a year and a half ago, consulted me respecting an excrescence on his tongue, which he informed me, notwithstanding the use of various remedies, still continued to become worse, and excited in his mind very uneasy apprehensions.

On examination, I perceived a fungous excrescence of a foul and sloughy appearance, occupying the middle and posterior part of the tongue. It was about an inch in breadth; its edges were thick, elevated, and irregular; and near its centre was a small opening, which, on introducing a probe, was found to penetrate to some depth into the substance of that organ. It was of a pale whitish colour, and rose about one-fourth of an inch above the surface of the tongue.

Though the complaint, by his own account, was of some months standing, he could not distinctly say when it commenced, but conceived it must have existed several weeks previous to his paying much attention to it, since he perfectly recollected, that during that space of time, and before fore he thought of consulting any practitioner, he had experienced a sense of uneasiness in the part, but had been so much occupied in executing orders, in the line of his business, as to call off his attention. This account seems extremely probable, since we know, from recent experiments, that the tongue is not endowed with that exquisite sensibility which it was formerly supposed to possess, and which we might be led a priori to conceive.

His deglutition was little, if at all, impeded by it; neither were any of the sublingual or maxillary glands apparently enlarged, though he alleged that one of the latter had been previously affected. It was not attended with much pain on pressure, but bled occasionally, when any rough or hard substance came into contact with it. The hæmorrhage, however, did not appear to have been either frequent or at any time profuse. The health and strength of the patient were evidently much impaired, owing probably to dyspeptic complaints, with which he had been for some time harrassed, as well as to the anxious state of his mind respecting the issue of the malady.

Mr. C. it must be observed, is of a scrophulous diathesis, and has, at various periods of his life, been subject to glandular swellings or obstructions in different parts of his body.

Under these circumstances he was put on a course of cicuta with calomel, from the latter of which remedies, in particular, when properly administered, and persisted in for a sufficient length of time, we had frequently witnessed the most beneficial effects, not only in ill-conditioned and inveterate ulcers, but also in glandular tumours or obstructions, whether of a scrophulous kind, or apparently approximating to a schirrous or carcinomatous nature. While country air and exercise were strictly enjoined, particular attention was also paid to obviate the deranged action of the digestive organs, and to procure a regular condition of the alvine discharge.

This plan, which was steadily pursued for some weeks, appeared at first to be productive of manifest advantage, for the health of the patient gradually improved under it, and the sore began to assume a more favourable aspect, while at the same time the excrescence indicated a tendency to separate and slough off. This, however, was but of short continuance, for the ulcer quickly again degenerated, and even assumed a more sordid appearance than before, spreading, and penetrating deeper into the substance of the tongue; the fungus also, which had acquired a dull leaden colour, became thicker and more elevated at the edges, and apparently less disposed to separate. In this state of things I was induced, by Mr. Carmichael's favourable reports of his success from the internal and external use of iron in some cases of supposed cancer, to make trial of this remedy. Accordingly, while the tinctura ferri muriati, and various other chalybeates, were prescribed internally, the carbonate of iron was employed externally under the following form.

℞. Pulv. tragacanth. comp. ℥ij. Carbonat. ferri ℥ß. Aq. rosar. q. s. ut fiat massa, in trochiscos, mediae magnitudinis, formanda. Quorum unus vel duo detineantur subinde superlinguam, donec liquescant.

Stimulant and detergent gargles were likewise occasionally used, with the view of exciting the action of the affected organ, and thereby of promoting the separation of the sloughs.

In a few days after adopting this plan, the patient being under the necessity of taking a journey to Liverpool, was ordered either to supply himself with a sufficient stock of his medicines, or to get them renewed occasionally during his absence. At this time the excrescence appeared as if beginning to separate from the sound parts beneath, and hopes were entertained that it would be soon entirely thrown off; the ulcer also, though somewhat deeper and more extensive, displayed a less unfavourable aspect.

In about three weeks he returned to town, when he informed me, with joy depicted on his countenance, that his health was greatly amended, and that his tongue was nearly well. From his account it appeared, that the excrescence had separated in successive layers or sloughs. On viewing the tongue, at this period, the surface, from which these had been cast off, as well as that of the ulcer, was covered with an opaque whitish crust. He was now ordered to discontinue the gargle, and confine himself to the use of the lozenges. With this injunction he cheerfully acquiesced, because, according to him, they alone had effected the favourable change. Within a few days afterwards, the crust dropped off, and left the parts beneath perfectly healed, and the cavity in the middle completely filled up.

Since the removal of his complaint, this patient has enjoyed better health than he recollects to have done for several years before.

Whether the present affection was of a carcinomatous or scrophulous nature, different sentiments may perhaps be entertained. The last of these two opinions is certainly countenanced by the marked predisposition of the patient to such affections, who, as already observed, had often laboured laboured under glandular swellings or obstructions in different parts of the body. This conclusion, respecting the nature of the disease, is perhaps still farther supported by the success of the mode of treatment pursued, which was in many respects similar to that commonly considered as most efficient in scrophula.

Some practitioners have, we are aware, maintained, that an affinity subsists between scrophula and carcinoma: but we cannot see any grounds for acquiescing in this opinion; since, whatever may be alleged to the contrary, no evidence has yet been produced to substantiate the assertion. Even, however, in this view of the subject, the publication of the present case cannot, we think, be deemed wholly unimportant, since, in as far as a conclusion may be drawn from a solitary instance, it affords additional evidence of the beneficial effects of iron in promoting the cicatrization of inveterate and ill-conditioned ulcers, and leads us to hope, that it may prove a valuable auxiliary in a class of diseases both dangerous and intractable.

On the whole, though we are inclined, as already mentioned, to doubt the specific powers of iron in genuine cancer, and are of opinion, that most of the cases brought forward by Mr. Carmichael are merely those of phagedenic ulceration, yet, as he also details one or two others, apparently of a carcinomatous nature, in which a cure was effected by its use, and as, moreover, it appears to have afforded relief in those cases wherein it did not ultimately succeed, these circumstances seem to us to render it worthy of the attention of practitioners. Hence we deprecate all hasty decision on the subject, and trust no one will rashly infer, because this remedy cannot accomplish every thing affirmed respecting it, that it is of no value; for were it only productive of relief in such a deplorable malady as cancer, that alone would be sufficient to entitle it to our regard, since, certainly it is no small advantage to be in possession of an agent capable of alleviating the severe pains of a disease, though we should still have to lament the want of one adequate to its cure.

In short, whatever may be the merits of the remedy under consideration, these, it is obvious, can only be ascertained by experiments made by different individuals accustomed to investigation, and unfettered by hypotheses or preconceptions of any kind.

Δόξα μὲν ἀνθρώποισι κακὸν μέγα, πεῖρα δ' ἄριστον.

*London, March* 1810.

